# Beyond Functional Outcomes: Patient-Reported Perioperative Experience After Implant Trapeziometacarpal Joint Arthroplasty for Thumb Osteoarthritis: A Multicenter Observational Study

**DOI:** 10.1016/j.jhsg.2026.101099

**Published:** 2026-08-07

**Authors:** Alice Mangeon, Laurent Obert, Daniel Lepage, François Loisel

**Affiliations:** ∗ Department of Orthopaedic, Trauma, Plastic and Reconstructive Surgery, Hand Trauma Unit (SOS Mains), SINERGIES Research Unit (UR 4662), University Marie and Louis Pasteur, Besançon University Hospital (CHU de Besançon), Besançon, France

**Keywords:** Patient-reported experience, Perioperative care, Satisfaction, Thumb carpometacarpal osteoarthritis, Trapeziometacarpal arthroplasty

## Abstract

**Purpose:**

Thumb carpometacarpal osteoarthritis is a common condition in older adults and may severely impair hand function and quality of life. When conservative treatment fails, trapeziometacarpal arthroplasty is a widely accepted surgical option, with well-documented clinical and functional outcomes. However, the subjective perioperative experience of patients remains poorly explored, despite its growing importance in value-based health care.

**Methods:**

This retrospective, multicenter observational study included patients who underwent primary implant trapeziometacarpal arthroplasty between November 2007 and December 2023 in two tertiary centers. A self-administered questionnaire specifically designed to assess perioperative experience was sent to all eligible patients. Demographic data, radiographic characteristics, patient satisfaction, perioperative discomfort, postoperative pain resolution, and time to “forgetting the thumb” were analyzed descriptively.

**Results:**

A total of 119 prostheses in 95 patients were analyzed, corresponding to a 42.6% response rate (95/223 eligible patients), with a mean follow-up of 6.4 ± 4.7 years. Overall satisfaction was high, with 83.2% of patients reporting being satisfied or very satisfied. Locoregional anesthesia was identified as the most unpleasant perioperative element by 22.7% of patients. Postoperative pain resolved rapidly in most cases, and 33.7% of patients reported “forgetting their thumb” within 4 months after surgery. Despite postoperative pain being frequently cited as unpleasant, only 2.5% of patients expressed a desire for improved analgesic protocols.

**Conclusions:**

Trapeziometacarpal arthroplasty is associated with high patient satisfaction and rapid perceived recovery. Beyond functional outcomes, perioperative experience particularly anesthesia-related discomfort and preoperative information plays a critical role in patient satisfaction. Integrating patient-reported experience into outcome assessment may help optimize perioperative care pathways.

**Type of study/level of evidence:**

Prognosis IV.

Thumb trapeziometacarpal (TMC) osteoarthritis (OA) is a prevalent degenerative condition, affecting approximately 13% to 26% of older adults, particularly postmenopausal women,^1^ and is highly variably experienced clinically despite an even higher radiographic prevalence. Clinically, it manifests as pain at the base of the thumb, reduced range of motion, decreased pinch strength, and progressive deformity**,** leading to substantial functional impairment and reduced quality of life. Initial management is conservative, including therapy, splinting, analgesics, and intra-articular injections. When these measures fail, surgical treatment may be proposed. Among the various surgical options, TMC joint arthroplasty has gained increasing popularity over the past two decades, supported by favorable clinical and functional outcomes reported in the literature.^2^

While surgical success has traditionally been evaluated using objective clinical scores and radiographic outcomes, there is growing recognition that patient-reported outcomes and experiences are essential components of care evaluation.^3^ Pain perception, perioperative anxiety, anesthesia experience, recovery expectations, and the subjective sense of functional normalization may substantially influence overall satisfaction, independent of objective outcomes.^4^ Previous studies in thumb CMC OA have increasingly emphasized pain, function, and patient-reported outcome measures (PROMs) as key end points following treatment. Several studies have also explored patient satisfaction and its determinants after surgery, highlighting that subjective outcomes may differ from objective clinical measures. However, most of the available literature focuses on postoperative functional results or satisfaction, while the detailed perioperative experience remains poorly characterized. Prior work suggesting that patient perceptions and expectations influence outcomes further supports the need to better understand the perioperative experience in this population.^5^, ^6^, ^7^

Despite the expanding use of PROMs, perioperative patient experience in TMC arthroplasty remains insufficiently documented. Understanding this experience may provide valuable insights to improve patient-centered care.

Since 2007, primary TMC arthroplasty has been routinely performed at our institutions using modular, retentive implants (ISIS until 2021, then HORUS; Evolutis). The aim of this study was to report patient-reported perioperative experiences and satisfaction following TMC arthroplasty.

## Materials and Methods

### Study design

This was a retrospective cross-sectional survey, with questionnaires administered at a single time point after surgery (mean follow-up: 6.4 ± 4.7 years), multicenter, observational study conducted at two institutions. This study was performed outside the scope of the French Jardé law and was registered under protocol number 2025/967.

### Patients

All patients who underwent primary TMC joint implant arthroplasty between November 2007 and December 2023 were eligible. Exclusion criteria included the following:

1. post-traumatic arthritis,
2. revision arthroplasty initially performed elsewhere,
3. associated procedures altering perioperative experience (eg, scapho trapezo trapezoid pyrocarbon implant and distal scaphoid resection),
4. use of implants other than ISIS or HORUS (semiconstrained prosthesis with screwed cup), and
5. nonresponse to the questionnaire.

Post-traumatic arthritis was defined as OA occurring after a documented history of prior trauma to the thumb CMC joint (eg, fracture, dislocation, or ligament injury).

Simultaneous bilateral procedures were not performed. However, some patients underwent staged bilateral arthroplasties at different time points (months to years apart), and each procedure was analyzed independently.

### Surgical procedure and perioperative management

All procedures were performed in an ambulatory setting under locoregional anesthesia. All procedures were performed under locoregional anesthesia. A tourniquet was applied at the upper arm level in all patients. Patients remained awake during the procedure. Patients were offered the possibility of watching the procedure on a screen if they wished. Postoperative analgesia consisted of level I and II analgesics, without the use of opioids or nonsteroidal anti-inflammatory drugs. Preoperative radiographs were independently reviewed by two readers (one senior and one junior) to determine disease staging. In case of disagreement, the senior reviewer’s assessment was retained.

### Assessment of perioperative experience

Patient experience was assessed using a self-administered, nonvalidated questionnaire specifically designed for this exploratory study, based on commonly reported domains of perioperative patient experience in surgical care. The questionnaire was developed by the surgical team involved in the perioperative management of TMC implant arthroplasty patients.

The questionnaire addressed is in Appendix 1, available online on the Journal’s website at https://www.jhsgo.org.

### Statistical analysis

Results are presented as means ± standard deviation or percentages as appropriate. Given the exploratory design of the study and the predominantly qualitative and categorical nature of the data derived from open-ended responses, results are presented using descriptive statistics (frequencies, percentages, and means ± standard deviation for continuous variables when appropriate). Formal assessment of normality was not deemed relevant in this context, as no comparative or inferential analyses were performed.

## Results

### Population

Among 223 contacted patients, 95 patients (119 prostheses) were included in the final analysis (Table 1). The mean age at surgery was 64.7 ± 8.4 years. Mean follow-up was 6.4 ± 4.7 years. Most patients were women (78.9%). Radiographically, 54.6% had isolated TMC OA, most commonly Dell stages II–III. Pantrapezial OA was present in 33.6%.

**Table 1:** Demographic Data and Preoperative Radiographic Data

Data	N (%)
Female	75 (78.9)
Heavy manual occupation	15 (15.8)
Manual hobbies	25 (26.3)
Operated side	
Dominant	43 (36.1)
Nondominant	43 (36.1)
Not reported	33 (27.7)
Implant type	
HORUS	37 (31.1)
ISIS	82 (68.9)
Implantation center	
Center 1	106 (89.1)
Center 2	13 (10.9)
Preoperative peritrapezial osteoarthritis	40 (33.6)
Dell classification	
1	5 (4.2)
2	27 (22.7)
3	38 (31.9)
4	4 (3.4)
Not reported	45 (37.8)
Presence of trapezial osteophytes	47 (39.5)

### Patient satisfaction

Overall satisfaction was high: 52.1% of patients were very satisfied and 31.1% satisfied. Only 5.9% reported being disappointed or very disappointed.

### Time to surgery

More than 40% of patients reported a time of 2–5 years between symptom onset and surgery (Tables 2 and 3). The main reasons were prior corticosteroid injection, personal constraints, and lack of information regarding surgical options.

**Table 2:** Patient-Estimated Time Between Onset of Pain and Surgery∗

Data	N (%)
<1 y	8 (6.7)
1–2 y	8 (6.7)
2–3 y	27 (22.7)
3–5 y	21 (17.6)
5–7 y	10 (8.4)
>10 y	5 (4.2)
Long but indeterminate duration	16 (13.4)
Does not remember	2 (1.7)
Not reported	22 (18.5)

∗ Values were categorized from open-ended patient responses. Responses classified as <1 year included “3–4 months,” “6 months,” and “several months.” Responses classified as 1–2 years included “1 year,” “15 months,” and “more than 1 year.” Responses classified as 2–3 years included “2 years” and “more than 2 years.” Responses classified as 3–5 years included “3 years,” “3–4 years,” and “4 years.” Responses classified as 5–7 years included “5 years,” “6 years,” and “6–7 years.” Responses classified as >10 years included “10 years,” “10–15 years,” “15 years,” and “20 years.” “Long but indeterminate duration” included qualitative responses such as “several years,” “a few years,” “years,” and “a long time.”

**Table 3:** Reasons Reported by Patients Explaining the Delay Between Pain Onset and Surgery∗

Data	N (%)
Corticosteroid injection(s)	27 (22.7)
Apprehension	5 (4.2)
Personal constraints	20 (16.8)
Lack of information (did not know surgery was possible)	23 (19.3)
Delay to obtain a surgical consultation	9 (7.6)
Thought the pain would resolve	8 (6.7)
Did not wait	1 (0.8)
Not reported	28 (23.5)

∗ For two procedures, patients reported two different reasons.

### Pain resolution and functional perception

Postoperative pain was described as “rapidly” resolving by 14.5% of patients, while 13.5% reported complete pain disappearance within 15 days (Tables 4 and 5). Importantly, 33.7% of patients reported “forgetting their thumb” within 4 months, reflecting early subjective functional normalization.

**Table 4:** Time to Disappearance of Pain After Surgery

Data	N (%)
≤1 wk	9 (7.6)
1–2 wk	7 (5.9)
3 wk	1 (0.8)
1 mo	15 (12.6)
2 mo	6 (5.1)
3 mo	5 (4.2)
4–5 mo	2 (1.7)
6 mo	4 (3.4)
8 mo	1 (0.8)
Immediate	13 (10.9)
Rapidly∗	17 (14.3)
A few weeks	4 (3.4)
A few months	1 (0.8)
Never	4 (3.4)
Does not remember	6 (5.1)
Not reported	24 (20.2)

∗ “Rapidly” corresponds to qualitative patient-reported responses such as “very quickly,” “rapidly,” or “almost instantaneously.” Responses categorized as ≤1 week included “1.5 days,” “2 days,” “72 hours,” “1 week,” and “the day after surgery.” Responses categorized as 1–2 weeks included “10 days,” “15 days,” and “2 weeks.”

**Table 5:** Time to “Forgetting the Thumb” After Surgery∗

Data	N (%)
≤8 d	2 (1.7)
15 d	6 (5.1)
2–3 wk	1 (0.8)
1 mo	10 (8.4)
2 mo	9 (7.6)
3–4 mo	12 (10.1)
6 mo	10 (8.4)
≥1 y	3 (2.5)
Immediate	4 (3.4)
Quickly	11 (9.2)
A few weeks	1 (0.8)
A few months	7 (5.0)
Never	13 (10.9)
Does not remember	3 (2.5)
Not reported	27 (22.7)

∗ Values were categorized from open-ended patient responses. Responses classified as ≤8 d included “no more than 1 week” and “8 days.” Responses classified as 15 days included “after suture removal” and “15 days.” Responses classified as 1 month included “4 weeks” and “1 month.” Responses classified as 3–4 months included “3 months” and “4 months.” Responses classified as ≥1 year included “1 year” and “1.5 years.”

### Perioperative discomfort

Locoregional anesthesia was the most frequently reported unpleasant element (22.7%), followed by postoperative pain (21.0%). Nevertheless, only a minority of patients expressed a desire to improve postoperative analgesic protocols (Table 6).

**Table 6:** Elements to Improve Reported by Patients

Data	N (%)
Nothing	59 (49.6)
Improve information/organization around locoregional anesthesia	5 (4.2)
Improve analgesic protocol at home	3 (2.5)
Inform about variable postoperative recovery time	3 (2.5)
Reduce operative delays	2 (1.7)
Perform general anesthesia	1 (0.8)
Provide more precise information about surgery time	1 (0.8)
Advise patients not to wait too long before surgery	1 (0.8)
Does not know	1 (0.8)
Not reported	43 (36.1)

## Discussion

This study demonstrates a high level of patient satisfaction following TMC arthroplasty, consistent with previous reports focusing on clinical and functional outcomes.^7^ However, its primary contribution lies in the detailed exploration of perioperative patient experience, an aspect rarely addressed in the context of thumb implant arthroplasty.

### Patient experience beyond functional outcomes

Several studies have highlighted discrepancies between surgeon-reported outcomes and patient perceptions, emphasizing the importance of PROMs in modern health care evaluation.^8^ Our findings reinforce the notion that technical success does not automatically equate to optimal patient experience. For future studies, patient involvement in the design of such questionnaires would be valuable to enhance their relevance and validity.

### Anesthesia in the perioperative experience

Locoregional anesthesia was the most frequently reported unpleasant perioperative element (22.7%), followed by postoperative pain (21.0%). Overall satisfaction with surgery remained high (83.2%). Similar findings have been reported in hand surgery, where anxiety, tourniquet discomfort, and environmental factors contribute to negative experiences.^9^

In this context, wide-awake local anesthesia no tourniquet (WALANT) anesthesia has gained increasing interest. Previous studies have shown comparable functional outcomes and improved perioperative comfort, particularly by eliminating tourniquet-related pain.^10^ Furthermore, lower anxiety levels have been reported with WALANT compared to general anesthesia.^11^ Recent studies have also reported the feasibility of WALANT anesthesia for thumb carpometacarpal (CMC) arthroplasty, with satisfactory perioperative experience and functional outcomes.^10^ These data suggest that anesthesia choice may directly influence patient experience and satisfaction.

### Subjective recovery and “forgetting the thumb”

The concept of “forgetting the thumb” represents a patient-centered outcome analogous to joint awareness described in lower limb arthroplasty. The proportion of patients reporting “forgetting the thumb” within the first postoperative months suggests progressive subjective functional normalization after TMC arthroplasty. However, given the retrospective design and the absence of a comparison group, no direct comparison with other CMC OA procedures can be made. While 33.7% of patients reported “forgetting their thumb” within 4 months, a majority of patients remained aware of their thumb beyond this time point, suggesting a variable and sometimes prolonged subjective recovery period.

### Time to surgery and care pathways

The prolonged time between symptom onset and surgery, often linked to insufficient information, highlights the need for improved communication between primary care physicians, surgeons, and patients. Early referral and education may reduce adverse symptom experiences and optimize outcomes. Importantly, this time interval should be interpreted in light of the fact that many individuals with radiographic evidence of CMC OA remain asymptomatic or manage their symptoms conservatively without progressing to surgery.

### Limitations

This study has several limitations, including its retrospective design, a substantial risk of recall bias, as patients were asked to retrospectively report their perioperative experience, and a substantial nonresponse rate. In particular, perioperative experiences may be influenced by memory decay and overall satisfaction with surgical outcomes (halo effect), potentially leading to an overestimation of positive perceptions. Moreover, the retrospective reporting of time-sensitive early postoperative outcomes (eg, pain resolution and time to “forgetting the thumb”) may be imprecise, as patients were required to recall specific timeframes several years after surgery. The response rate below 50% also represents a significant limitation and may introduce substantial nonresponse bias, as responders and nonresponders may differ in terms of satisfaction, complications, or long-term perception of surgical outcomes, thereby limiting the generalizability of the findings. In addition, the use of a nonvalidated questionnaire may affect the reliability of the findings, and the retrospective assessment of acute perioperative experiences represents a methodological limitation inherent to the study design.

However, this instrument was specifically developed to explore clinically relevant aspects of perioperative experience that are not captured by existing PROMs.

Despite these limitations, the exploratory nature of the study and the consistency of the results support the relevance of the findings.

This study provides exploratory insights into patient-reported perioperative experience after TMC arthroplasty. While satisfaction was high, several factors, particularly anesthesia-related discomfort and preoperative information, appeared to influence patient perception. Further prospective studies combining validated patient-reported experience measures with qualitative approaches may help better characterize perioperative patient experience after TMC arthroplasty.

## Conflicts of Interest

François Loisel, MD, PhD, has financial or association involvement with Evolutis and Medartis. No benefits in any form have been received or will be received by the other authors related directly to this article.
