# Supplementary material for: Beyond Functional Outcomes: Patient-Reported Perioperative Experience After Implant Trapeziometacarpal Joint Arthroplasty for Thumb Osteoarthritis: A Multicenter Observational Study
Source: J Hand Surg Glob Online. 2026 Aug 7;8(6):101099. doi: 10.1016/j.jhsg.2026.101099 (PMC13476607; doi:10.1016/j.jhsg.2026.101099)
Supplement: Supplementary file 1 [file mmc1.docx]

Appendix 1 : Questionnaire intended to patients

How long did you suffer before having surgery?

Why did you wait so long ?

Today, you are :

- Very disappointed (you would not have this operation again)
- Disappointed (you expected better)
- Satisfied (it’s exactly what you expected)
- Very satisfied (it’s better than you hoped)

After the procedure, what is the minimum time it took for the pain to disappear?

After the procedure, how long did it take before you “forgot” about your thumb?

What was the most unpleasant moment surrounding this procedure ?

What should the surgeon say or do to better prepare you for the procedure ?
